# PIERCE1 is critical for specification of left-right asymmetry in mice

**DOI:** 10.1038/srep27932

**Published:** 2016-06-16

**Authors:** Young Hoon Sung, In-Jeoung Baek, Yong Hwan Kim, Yong Song Gho, S. Paul Oh, Young Jae Lee, Han-Woong Lee

**Affiliations:** 1 Department of Biochemistry, College of Life Science and Biotechnology and Yonsei Laboratory Animal Research Center, Yonsei University, Seoul 03722, Republic of Korea; 2 Department of Physiology and Functional Genomics, College of Medicine, University of Florida, Gainesville, FL 32610, USA; 3 Pohang University of Science and Technology, Pohang 37673, Republic of Korea; 4 Lee Gil Ya Cancer and Diabetes Institute, Gachon University, Incheon 21999, Republic of Korea

## Abstract

The specification of left-right asymmetry of the visceral organs is precisely regulated. The earliest breakage of left-right symmetry occurs as the result of leftward flow generated by asymmetric beating of nodal cilia, which eventually induces asymmetric Nodal/Lefty/Pitx2 expression on the left side of the lateral plate mesoderm. PIERCE1 has been identified as a p53 target gene involved in the DNA damage response. In this study, we found that *Pierce1*-null mice exhibit severe laterality defects, including *situs inversus totalis* and heterotaxy with randomized *situs* and left and right isomerisms. The spectrum of laterality defects was closely correlated with randomized expression of *Nodal* and its downstream genes, *Lefty1/2* and *Pitx2*. The phenotype of *Pierce1*-null mice most closely resembled that of mutant mice with impaired ciliogenesis and/or ciliary motility of the node. We also found the loss of asymmetric expression of Cerl2, the earliest flow-responding gene in the node of *Pierce1*-null embryos. The results suggest that *Pierce1*-null embryos have defects in generating a symmetry breaking signal including leftward nodal flow. This is the first report implicating a role for PIERCE1 in the symmetry-breaking step of left-right asymmetry specification.

The left-right (LR) asymmetric pattern of the visceral organs, including the lung, heart, stomach, and spleen, is conserved across mammalian species. Mice have a lung with four right lobes and one left lobe, a left-pointing heart, and a left-sided stomach and spleen. The normal specification of LR asymmetry of the visceral organs is called *situs solitus* (SS). In contrast, *situs inversus totalis* (SI) is a malformation in which the specification of LR asymmetry of the visceral organs is completely reversed. Loss of LR specification also results in heterotaxy with aberrant positioning of visceral organs and isomerism of normally asymmetric organs^1^,^2^,^3^.

Because sonic hedgehog (SHH) and NODAL were first identified as left-right determinants in chicks approximately 20 years ago^4^, numerous other genes have been shown to be involved in the four steps required for specification of LR asymmetry^5^,^6^. The first step requires symmetry breaking, which is mediated by leftward flow in the node that results from the posterior tilt of the rotation axis of nodal cilia^7^,^8^. The absence or immotility of nodal cilia has been reported in several mutant strains of mice that exhibit abnormal LR asymmetry^9^,^10^,^11^,^12^,^13^. One of them is *iv*/*iv* (*inversus viscerum*) mutant mice, which display diverse patterns of laterality, including SS, SI, and left and right isomerisms^14^,^15^,^16^. Immotility of nodal cilia in the *iv*/*iv* mutant strain is caused by a missense mutation on the *iv* gene, which encodes dynein axonemal heavy chain 11 (DNAHC11), an active component of a nodal cilium^17^,^18^.

The second step of LR asymmetry specification involves the transfer of asymmetric signal(s) to the left side of the lateral plate mesoderm (LPM). According to two cilia model, non-motile polycystin-2 containing cilia of the perinodal cells (crown cells) recognizes nodal flow generated by motile cilia at the node and initiates an asymmetric calcium signal at the left border of the node^19^. NODAL is a member of the transforming growth factor-β (TGF-β) superfamily and plays a crucial role in transferring the node signal to the left LPM^20^,^21^. NODAL produced in the perinodal cells may be directly transported to the left LPM^20^. The long-range action of NODAL needs to form heterodimers with growth-differentiation factor 1 (GDF1), another member of the TGF-β superfamily^22^. Although the *Nodal* gene is bilaterally transcribed in the perinodal cells, high levels of active NODAL in the left side of the node are achieved by asymmetric expression of the NODAL antagonist, CERL2, in the right side of the node^5^,^23^,^24^.

The third step requires asymmetric expression of Nodal and Lefty2 in the left LPM. Transported NODAL activates its own expression in the left LPM through a NODAL-responsive asymmetric enhancer (ASE) of the *Nodal* gene^25^,^26^. Lefty2 expression is also induced through the ASE by locally produced NODAL in the left LPM^27^. LEFTY1 on the left side of the midline induced by NODAL^28^ may function as a barrier to maintain the expression of Nodal and Lefty2 in the left LPM. The expression of Nodal and Lefty is dynamically regulated by the positive and negative feedback loops mediated by NODAL and LEFTY1/2^25^,^26^,^27^.

The last step of LR asymmetry specification is *situs*-specific organogenesis. The key regulator of this step is *Pitx2*, which encodes a transcription factor with zinc finger motifs. PITX2 is expressed asymmetrically in the left LPM, and its expression is regulated by NODAL signaling^29^,^30^.

Retinoblastoma (Rb)/E2F transcription factors and p53 are integrated into a network that is critical for tumor suppression, and they mutually regulate each other through p16^Ink4a^, p21^Waf1^, and p19^Arf^^31^. Both p53 and Rb/E2F have critical roles in suppressing tumor initiation and progression by controlling a plethora of genes. p53-induced expression in *Rb*^−/−^ cells 1 (*Pierce1*) was first identified as an upregulated gene in *Rb*^−/−^ mouse embryonic fibroblasts (MEFs)^32^. Pierce1 shows a cell cycle-dependent expression pattern and is upregulated in S and/or G2, and its expression is significantly down-regulated by spontaneous immortalization of *Rb*^−/−^ MEFs^32^. Through subsequent analyses, we demonstrated that *Pierce1* is not directly regulated by Rb/E2F, but is a novel p53 target gene^33^. Pierce1 is involved in regulating the DNA damage response, and its knockdown attenuates the expression of diverse p53 target genes upon ultraviolet C (UVC) irradiation^33^. This evidence indicates that Pierce1 plays a critical function in the regulation of cellular transformation and tumorigenesis. To analyze the physiological role of Pierce1, we generated a mouse model deficient for Pierce1 and unexpectedly observed interesting developmental defects. The mice exhibited diverse patterns of laterality, including SS, SI, and left and right isomerisms. We also found randomized expression of asymmetric genes, such as *Nodal*, *Lefty2*, and *Pitx2*, in the LPM of *Pierce1*-null embryos, indicating that PIERCE1 functions at the initial step of LR specification. This is the first report to identify PIERCE1 as one of the key regulators of LR asymmetry specification.

## Results and Discussion

### *Pierce1* ^Gt^ is a null allele

We previously reported that *Pierce1* is a downstream target of p53 and may play an important role in maintaining genomic integrity against genotoxic stresses^33^; however, the *in vivo* role of *Pierce1* has yet to be elucidated. To investigate the function of PIERCE1 *in vivo*, *Pierce1*-deficient mice were generated using an embryonic stem (ES) cell clone harboring a *g*ene-trap cassette in intron 2 of the *Pierce1* locus (*Pierce1*^Gt^, Supplementary Fig. S1A). Mice homozygous for the gene-trapped *Pierce1* allele (*Pierce1*^Gt/Gt^) were obtained by intercrossing heterozygotes (Supplementary Fig. S1B). Whereas *Pierce1* expression is prominent in several tissues, including the brain, lung, kidney, and testis^32^, *Pierce1* transcripts were undetectable in these tissues in *Pierce1*^*Gt/Gt*^ mice by reverse transcriptase (RT)-PCR (Supplementary Fig. S1C), indicating that the *Pierce1*^Gt^ allele is a null allele of *Pierce1*.

### PIERCE1 deficiency results in randomization of body *situs*

**Figure 1:**
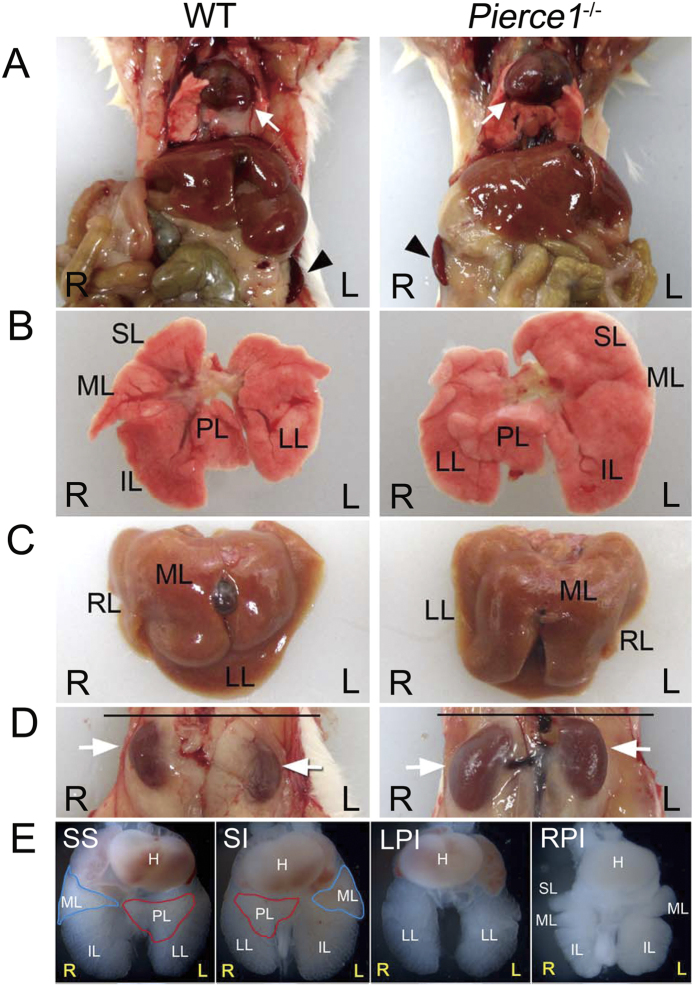
Diverse laterality phenotypes in *Pierce1*^−/−^ mice. (**A**) The SI *Pierce1*^−/−^ mice show dextrocardia and right-sided spleens. Arrow and arrowhead indicate the heart apex and the spleen, respectively. (**B**,**C**) Mirror-imaged inversion of the lobulation patterns of the lung (**B**) and the liver (**C**) in SI *Pierce1*^−/−^ mice. The normal lung is bilaterally asymmetric with one left lobe (LL) and four right lobes (superior [SL], middle [ML], inferior [IL], and postcaval [PL] lobes). SI embryos exhibit a mirror image of the normal lung pattern. (**D**) Reversed rostral-caudal arrangement of the kidneys in SI *Pierce1*^−/−^ mice. Arrows indicate the positions of right and left kidneys. (**E**) Lung lobation patterns (ventral views) of E14.5 *Pierce1*^−/−^ embryos with SS, SI, bilateral uni-lobed lungs (LPI), and bilateral tetra-lobed lungs (RPI). Approximately half of the *Pierce1*^−/−^ embryos had LPI or RPI. Red and blue lines indicate postcaval (PL) and middle (ML) lobes, respectively. IL, inferior lobe.

Of 169 offspring obtained from intercrosses between *Pierce1*^+/−^ mice, 52 mice (31%) were wild-type (WT), 95 mice (56%) were *Pierce1*^+/−^, and 22 mice (13%) were *Pierce1*^−/−^ (Supplementary Table S1), which deviates significantly from the expected Mendelian ratio (Chi square *P* = 0.0013). Because neonatal lethality was not evident, these results indicate that *Pierce1*^−/−^ mice are partially embryonic lethal. The surviving *Pierce1*^−/−^ mice appeared to be grossly normal, viable, and fertile. Upon autopsy, however, we found striking *situs* alterations in *Pierce1*^−/−^ mice (Fig. 1A–D). Among 78 *Pierce1*^−/−^ adults examined, 44 mice (59%) showed SS and 32 (41%) showed SI. SI mutants exhibited complete mirror-image reversal of the position of the heart, stomach, spleen (Fig. 1A), and kidney (Fig. 1D) along the LR axis. The lobation patterns of the lungs (Fig. 1B) and liver (Fig. 1C) were reversed as well. This phenotype demonstrates that PIERCE1 is an important regulator of LR specification.

### PIERCE1 deficiency results in partial embryonic lethality associated with heterotaxia

As the number of *Pierce1*^−/−^ mice generated from heterozygous intercrosses was much less than the expected Mendelian ratio (Supplementary Table S1), we examined the possibility that *Pierce1*^−/−^ mice were embryonic lethal. Most of the E13.5 and E14.5 *Pierce1*^−/−^ embryos exhibited grossly normal appearance and were recovered at the expected Mendelian ratio (Chi square *P* = 0.5246), but 10 out of 30 *Pierce1*^−/−^ embryos at E14.5 were found dead (Supplementary Table S1). Interestingly, *Pierce1*^−/−^ embryos at E13.5 and E14.5 exhibited a wide spectrum of laterality defects, including SI and left and right isomerisms (Fig. 1E). SS embryos had normal lungs with one left lobe and four right lobes, whereas the lungs of SI embryos had a mirror image of the normal lung pattern. Most of the dead mutant embryos exhibited bilateral uni-lobed lungs (left pulmonary isomerism, LPI; Fig. 1E) or bilateral tetra-lobed lungs (right pulmonary isomerism, RPI; Fig. 1E) with defects, such as persistent truncus arteriosus in great arteries (Supplementary Fig. S2).

To analyze LR asymmetry defects in detail, 20 E13.5 *Pierce1*^−/−^ embryos were examined using micro-computed tomography (μCT). *Pierce1*^−/−^ embryos displayed SS (9/20), SI (2/20), and various forms of heterotaxy (9/20), including LPI and RPI, with associated cardiac anomalies (Supplementary Table S2). While the apex of the heart pointed leftward in all wild-type embryos, the cardiac apex in *Pierce1*^−/−^ mice pointed to the left (12/20), the right (dextrocardia, 4/20), or the midline (mesocardia, 4/20) (Supplementary Table S2 and Supplementary Fig. S3). Most embryos with SI or heterotaxy had various cardiac anomalies, such as interventricular septal defects, hypoplastic heart defects, mitral valve defects, atrophy of the left ventricle, and dilated atria. Laterality defects of the lung lobation pattern were also observed in mutant embryos (Supplementary Table S2 and Supplementary Fig. S3). Among nine mutant embryos with heterotaxy, three had bilateral tetra-lobed lungs (RPI), three had bilateral uni-lobed lungs (LPI), and one had a normal lung lobation pattern without a postcaval lobe (Supplementary Table S2 and Supplementary Fig. S3). We found that the inferior vena cava was reversed (to the left side) in four heterotaxy mutants, as well as in all SI mutants (Supplementary Table S2). Reverse positioning of the stomach (to the right side of the abdomen) was found in some heterotaxy mutants as well as SI mutants (Supplementary Table S2 and Supplementary Fig. S3). Taken together, these data indicate that the partial embryonic lethality observed in *Pierce1*^−/−^ mice is closely associated with heterotaxy.

### Randomized expression of the asymmetric genes, *Nodal*, *Lefty1/2*, and *Pitx2* in *Pierce1* ^−/−^ embryos

**Figure 2:**
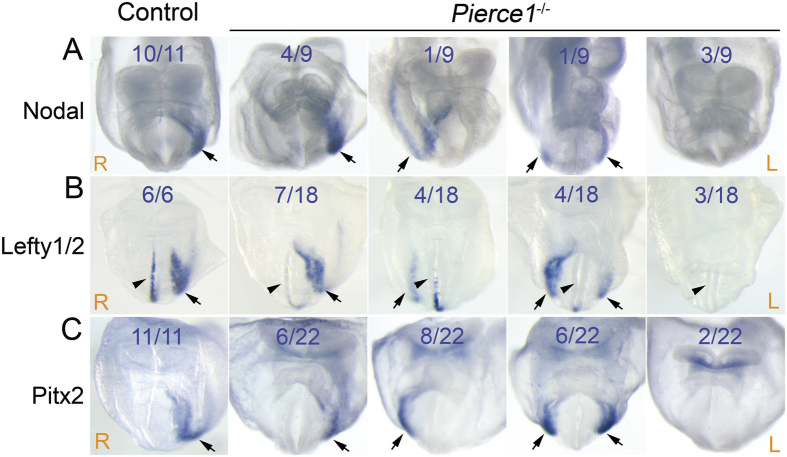
Expression of Nodal, Lefty1/2, and Pitx2 in control and *Pierce1*^−/−^ embryos. (**A**–**C**) The expression patterns of Nodal (**A**), Lefty1/2 (**B**), and Pitx2 (**C**) in E8.0 control and *Pierce1*^−/−^ embryos were examined by whole-mount *in situ* hybridization using antisense riboprobes. Expression of the asymmetric genes was randomized in *Pierce1*^−/−^ embryos. The ratios indicate the numbers of embryos exhibiting each expression pattern. Arrows indicate the detection of transcripts at the LPM. Note down-regulation of Lefty1 expression (arrowheads) in *Pierce1*^−/−^ embryos. The orientation of the embryos is indicated by R (right) and L (left).

The specification of LR asymmetry is determined by the expression of *Nodal* and its downstream genes, *Lefty1*, *Lefty2*, and *Pitx2*, in the LPM during early embryonic stages^6^. Nodal expression is detected in the perinodal cells and on the left side of the LPM, Lefty1 is expressed on the left side of the midline, and Lefty2 and Pitx2 are found in the left LPM of wild-type mouse embryos^6^. These asymmetric gene expression patterns were observed in both wild-type and *Pierce1*^+/−^ embryos (Fig. 2). Although there was no noticeable difference in Nodal expression in the perinodal cells in *Pierce1*^−/−^ embryos (n = 11; Supplementary Fig. S4), Nodal expression in the LPM was randomized in *Pierce1*^−/−^ embryos: normal (left side, 4/9), inverted (right side, 1/9), bilateral (both sides, 1/9), or absent (neither side, 3/9) (Fig. 2A). Lefty1 expression was down-regulated in the midline and Lefty2 expression was detected on the left side (7/18), right side (4/18), both sides (4/18), and neither side (3/18) of the LPM in *Pierce1*^−/−^ embryos (Fig. 2B). Likewise, Pitx2 expression was also randomized: on the left (6/22), right (8/22), both (6/22), and neither (2/22) side of the LPM (Fig. 2C). Left- and right-sided expression of the asymmetric genes in *Pierce1*^−/−^ mice may represent SS and SI, respectively. Conversely, bilaterally present or absent expression of these genes in *Pierce1*^−/−^ embryos may correspond to left or right isomerisms, respectively.

The randomized expression of asymmetric genes suggests that PIERCE1 functions during the initial step(s) of LR specification in the node. The expression of *Pierce1* in E8.0 embryos was determined by whole-mount *in situ* hybridization using a *Pierce1* riboprobe. We observed relatively strong expression of *Pierce1* in the node area (Supplementary Fig. S5), suggesting that PIERCE1 functions at the node for LR specification. Based on our results, PIERCE1 is likely involved in the earliest step(s) of LR asymmetry determination, such as symmetry breaking at the node.

Among numerous mutant mouse models that manifest laterality defects (Supplementary Table S3), only a few of them (*Arl13b*^−/−^, *Dnahc5*^−/−^, *iv/iv*, *Noto*^−/−^, *Rfx3*^−/−^, and *Zic3*^−/−^) exhibit the full spectrum of laterality phenotypes shown in *Pierce1*^−/−^ mice^18^,^34^,^35^,^36^,^37^,^38^. Randomized expression of Nodal in the LPM of *Arl13b*^−/−^, *iv*/*iv*, *Noto*^−/−^, and *Zic3*^−/−^ embryos and biased bilateral expression in *Rfx3*^−/−^ embryos have been reported^34^,^36^,^37^,^38^,^39^. Interestingly, all of these mutants have defects in either ciliogenesis and/or ciliary motility: 1) short cilia (*Arl13b*^−/−^, *Noto*^−/−^, *Rfx3*^−/−^, and *Zic3*^−/−^)^36^,^37^,^40^,^41^, 2) disorganized alignment of cilia (*Dnahc5*)^35^, and 3) rigid and immotile cilia (*iv/iv*)^18^. ARL13B is a small regulatory GTPase involved in ciliogenesis^40^. NOTO is an essential regulator of multiple genes involved in ciliogenesis and ciliary motility, including *Dnahc11* (iv), *Dnahc5*, and *Nphp3* via the Foxj1 and Rfx3 transcription factors^36^.

**Figure 3:**
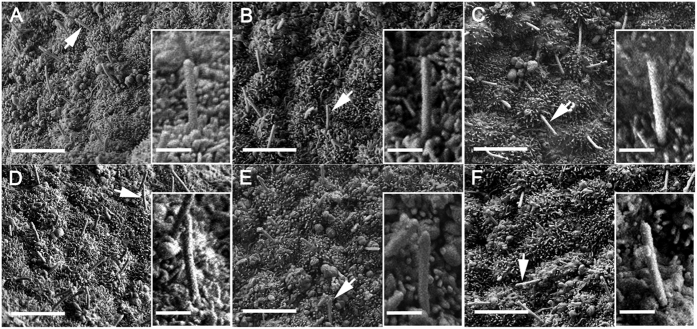
Lack of monocilia malformation on the nodes of E7.5 *Pierce1*^−/−^ embryos. (**A**–**F**) Scanning electron micrographs of embryonic nodes of WT (**A**) and representative *Pierce1*^−/−^ (**B**–**F**) embryos. There is no obvious ciliary malformation, such as duplication, bifurcation, partial bifurcation, or bulging in the nodes of E7.5 *Pierce1*^−/−^ embryos (n = 14). A cilium indicated by an arrow in each panel is enlarged in the box. Scale bars: (**A**–**F**), 5 μm; enlarged images, 1 μm.

**Figure 4:**
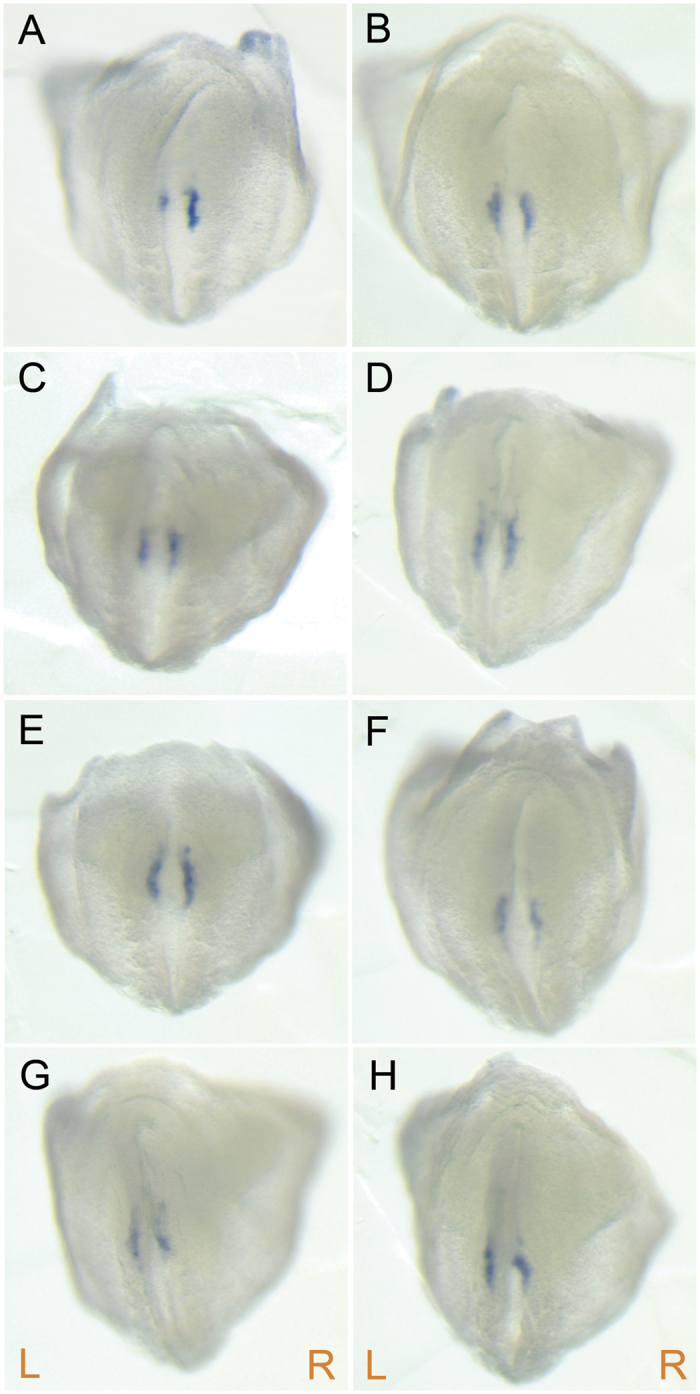
Loss of asymmetric expression of Cerl2 in the node of *Pierce1*^−/−^ embryos. (**A**–**H**) The expression patterns of Cerl2 in the node of control and *Pierce1*^−/−^ embryos with 3~5 somites were examined by whole-mount *in situ* hybridization using the *Cerl2* antisense riboprobe. (**A**) In control embryos, the Cerl2 expression on the left-side of the node is down-regulated. (**B**–**H**) The right-side dominant expression of Cerl2 is not clearly observed in *Pierce1*^−/−^ embryos (n = 7). All images are rear views. The orientation of the embryos is indicated by R (right) and L (left).

To investigate if ciliogenesis is affected in *Pierce1*^−/−^ embryos, the nodes of E7.5 embryos were examined with scanning electron microscopy (SEM). We found no malformations of monocilia development, such as duplication, bifurcation, partial bifurcation, bulging, or disorganized alignment in the nodal cells of E7.5 *Pierce1*^−/−^ embryos (Fig. 3). Next, we examined if expression of Dnahc11 (iv) and Noto is affected in *Pierce1*^−/−^ embryos (n = 5 for Dnahc11; n = 13 for Noto). Expression of both genes was detected at the node of E8.0 *Pierce1*^−/−^ embryos, comparable to the levels detected in wild-type controls (Supplementary Fig. S6). As the earliest responding gene to leftward flow in the node^23^,^24^, Cerl2 is bilaterally expressed in the perinodal cells at the early headfold stage when leftward nodal flow is locally generated. The local flow is sufficient to induce down-regulation of Cerl2 on the left-side at the late headfold stage^24^. We observed right-side dominant expression of Cerl2 in the node of WT and *Pierce1*^+/−^ embryos, while bilateral expression of Cerl2 was detected in *Pierce1*^−/−^ embryos (Fig. 4). These results strongly support that Pierce1 plays a critical role in generating a symmetry breaking signal including leftward nodal flow.

In this study, we uncovered the role of PIERCE1 in the regulation of LR asymmetry. *Pierce1*^−/−^ mice at mid-gestational periods were recovered in the expected Mendelian ratio and exhibited a wide spectrum of laterality defects, including isomerisms. In contrast, the mice presented at postnatal periods deviated significantly from the expected Mendelian ratio and only exhibited SS and SI without isomerisms. These results indicate that the mice with isomerisms die *in utero* due to various cardiovascular malformations associated with isomerisms. The laterality phenotypes of *Pierce1*^−/−^ mice suggest that PIERCE1 plays a pivotal role in determining the LR axis at the symmetry-breaking stage. The phenotype of *Pierce1*^−/−^ mice most closely resembles that of mutant mice with impaired ciliogenesis and/or ciliary motility of the node. Especially, morphologically normal nodal cilia and bilateral expression of Cerl2 in the node of *Pierce1*^−/−^ embryos suggest that *Pierce1*^−/−^ has defects in generating a symmetry breaking signal including leftward nodal flow.

Even if the expressional difference of Cerl2 existed between the left and right sides of the node in *Pierce1*^−/−^ embryos, the difference was very minor compared with that in controls. Thus, Nodal activity was expected to be suppressed by Cerl2 in the both side of the node. However, we observed randomized expression of *Nodal*, *Lefty2*, and *Pitx2* in the LPM (Fig. 2). Possible interpretation of this discrepancy is that the amount of bilaterally expressed CERL2 in *Pierce1*^−/−^ embryos is around threshold level to suppress NODAL activity, and thus the level of CERL2 in some *Pierce1*^−/−^ embryos is not sufficient to suppress NODAL activity in one or both side of the node. Nodal expression in the LPM is initially activated by transported NODAL protein from the node through the NODAL-responsive asymmetric enhancer of the *Nodal* gene, and then it is regulated through positive and negative feedback loops by NODAL itself and LEFTY2, respectively^25^,^26^. In *Pierce1*^−/−^ embryos, incomplete suppression of NODAL activity could initiate positive feedback loop for Nodal expression in the LPM. This discrepancy is also observed in *Arl13b*^−/−^ embryos^34^. Five out of six *Arl13b*^−/−^ embryos with 4–5 somites have bilateral expression of Cerl2, while Nodal expression is detected on the left side (5/19), right side (3/19), or both sides (11/19)^34^.

Notable expression of *Pierce1* is detected in adult tissues including the brain, lung, kidney and testis as well as in the node of E8.0 embryos (Supplementary Fig. S5)^32^. In fact, only distinct cells in these tissues have potential to generate motile cilia and flagella, implying that a dedicated genetic program controls gene expression for formation of motile cilia and flagella^42^. Previously we showed that *Pierce1* knockdown compromises the transcriptional activation of several p53 target genes upon UVC irradiation^33^. In addition, the hemagglutinin (HA)-tagged PIERCE1 protein was localized in the nucleus as well as in the cytoplasm (Supplementary Fig. S7). Based on these lines of evidence, we speculate that PIERCE1 contributes to the transcriptional control of genes involved in ciliogenesis and/or ciliary motility in the node although any functionally-defined protein domain of PIERCE1 has not been defined yet.

Further investigation regarding the biochemical, molecular, and genetic mechanisms by which PIERCE1 interacts with known factors involved in LR specification would elucidate the precise role of PIERCE1 during LR asymmetry specification.

## Methods

### Generation of mice carrying targeted mutations in the *Pierce1* gene

A *Pierce1*-deficient mouse model was generated using a gene-trapped mouse ES cell clone (Cell Line ID: OST3440, Lexicon Genetics, Inc.). The male chimeras were bred with C57BL/6J females, and germline F1 mice were bred with mice on a C57BL/6J or FVB/N background. In this study, most of the adult mice and embryos had mixed genetic backgrounds. The targeted allele was confirmed by PCR analysis. A three-primer PCR strategy was conducted to genotype mice and embryos using the following primers: 5′-CGAAGGCCAATTAGTGAAGTCAAGC-3′ as a common primer (C), 5′-CCAGAGAACAGGACTAAGAAGCACG-3′ as a wild-type-specific primer (WT), and 5′-ATAAACCCTCTTGCAGTTGCATC-3′ for the gene-trap allele-specific primer (GT) (Supplementary Fig. S1A). To detect *Pierce1* transcripts, complementary DNA (cDNA) samples were prepared using total RNAs from the brain, lung, kidney, and testis of control and *Pierce1*^−/−^ mice as previously described^32^, and RT-PCR was conducted using primer pairs specific for *Pierce1* (5′-CCAGTAACCAAACCTACGGA-3′ and 5′-AGTGGGTGATGTGATTGTCA-3′) and *Gapdh* (5′-ATCACTGCCACTCAGAAGAC-3′ and 5′-CACCACCTTCTTGATGTCATC-3′). All animal experiments were performed in accordance with the Korean Food and Drug Administration (KFDA) guidelines. Animal protocols were reviewed and approved by the Institutional Animal Care and Use Committees (IACUC) of Yonsei University (approval reference number, 2007-0004). All mice were maintained in a specific pathogen-free facility at the Laboratory Animal Research Center at Yonsei University.

### Whole-mount *in situ* hybridization

To study the expression patterns of *Cerl2*, *Dnahc11, Lefty1/2, Nodal, Noto, Pierce1*, and *Pitx2* in E8.0 ~ E8.5 embryos, whole-mount *in situ* hybridization was performed as previously described^43^. Antisense RNA probes were produced with template DNAs of *Cerl2*^34^, *Dnahc11*^19^, *Lefty1/2*^44^, *Nodal*^45^, *Noto* (NM_001007472, nt682-1181)*, Pierce1* (IMAGE clone: 317678), and *Pitx2*^46^ using a digoxigenin-UTP labeling kit (Roche) according to manufacturer’s instructions.

### Scanning electron microscopy (SEM)

To analyze node cilia formation, E7.5 embryos were removed from their extraembryonic tissues, fixed in 2.5% paraformaldehyde/2.5% glutaraldehyde/0.1 M Sorenson’s phosphate buffer for at least 24 h, and cut at informative planes with a fine scalpel blade. Embryos were post-fixed with osmium tetroxide, dehydrated with a graded series of ethanol, and critical point dried with liquid CO_2_. Embryos were mounted onto double-sided adhesive tape on metal stubs, coated with gold/palladium, and photographed with a JEOL high-resolution scanning electron microscope (JSM-7401F).

### Statistical Analysis

To determine the embryonic lethality of *Pierce1*^−/−^ mice from heterozygous matings, Chi-square analysis was conducted to compare with expected Mendelian ratio using chi square calculator (GraphPad QuickCalcs, http://graphpad.com/quickcalcs/chisquared1.cfm).

## Additional Information

**How to cite this article**: Sung, Y. H. *et al*. PIERCE1 is critical for specification of left-right asymmetry in mice. *Sci. Rep*. **6**, 27932; doi: 10.1038/srep27932 (2016).

## Supplementary Material

Supplementary Information
