# Supplementary material for: PIERCE1 is critical for specification of left-right asymmetry in mice
Source: Sci Rep. 2016 Jun 16;6:27932. doi: 10.1038/srep27932 (PMC4917697; doi:10.1038/srep27932)
Supplement: Supplementary Information [file srep27932-s1.pdf]

PIERCE1 is critical for specification of left-right asymmetry in mice

Young Hoon Sung,[1] In-Jeoung Baek,[1] Yong Hwan Kim,[2] Yong Song Gho,[3] S. Paul Oh,[2] Young Jae Lee,[4,*] Han-Woong Lee[5,*]

1Department of Convergence Medicine, University of Ulsan College of Medicine and Asan Institute for Life Sciences, Asan Medical Center, Seoul 05505, Republic of Korea.
2Department of Physiology and Functional Genomics, College of Medicine, University of Florida, Gainesville, FL 32610, USA.
[3]Pohang University of Science and Technology, Pohang 37673, Republic of Korea.
[4]Lee Gil Ya Cancer and Diabetes Institute, Gachon University, Incheon 21999, Republic of Korea.
[5]Department of Biochemistry, College of Life Science and Biotechnology and Yonsei Laboratory Animal Research Center, Yonsei University, Seoul 03722, Republic of Korea.
***Co-corresponding authors**

Supplementary Information

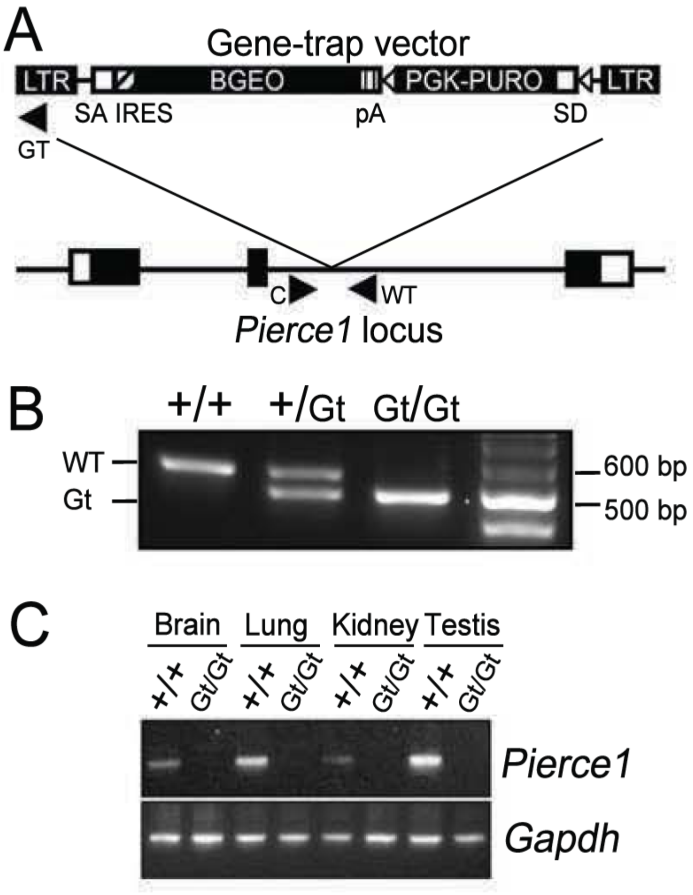

Supplementary Figure S1. Generation of *Pierce1*-deficient mice. (A) Gene-trap strategy. An ES cell clone with a retroviral insertion of a gene-trap vector in intron 2 of the *Pierce1* gene was used to generate the $Pierce1^{Gt}$ mouse line. The gene-trap vector consists of two long terminal repeat sequences (LTRs), splice acceptor (SA) and donor (SD) sites, an internal ribosome entry site (IRES), a β-galactosidase and neomycin-resistant fusion gene (BGEO), a polyadenylation signal (pA), and a puromycin-resistant cassette (PGK-PURO). Open triangles indicate *loxP* sequences. Exons are represented by boxes in the *Pierce1* locus. The coding region is indicated by black boxes. Primers for multiplex PCR genotyping are represented by filled triangles. C, common primer; WT, wild-type-specific primer; GT, gene-trap allele-specific primer. **(B)** A representative multiplex PCR genotyping result of wild-type (+/+), $Pierce1^{+/Gt}$, and $Pierce1^{Gt/Gt}$ mice. Upper and lower bands indicate wild-type (WT) and gene-trap (Gt) alleles, respectively. **(C)** RT-PCR analysis of *Pierce1* using total RNAs isolated from the brain, lung, kidney, and testis of wild-type (+/+) and $Pierce1^{Gt/Gt}$ mice. Glyceraldehyde 3-phosphate dehydrogenase (*Gapdh*) was used as an internal control.

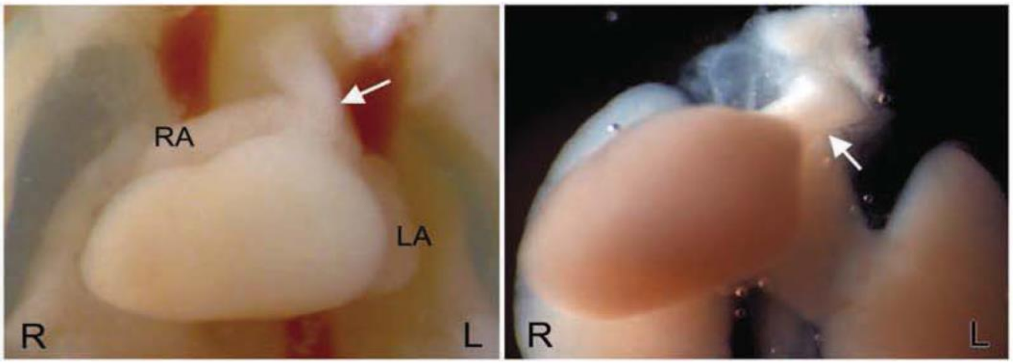

Supplementary Figure S2. **Persistent truncus arteriosus (PTA) and dextrocardia found in E14.5 *Pierce1*$^{-/-}$ embryos.** Arrows indicate outflow tract undivided by the aorta and pulmonary artery. RA, right atrium; LA, left atrium; R, right; L, left.

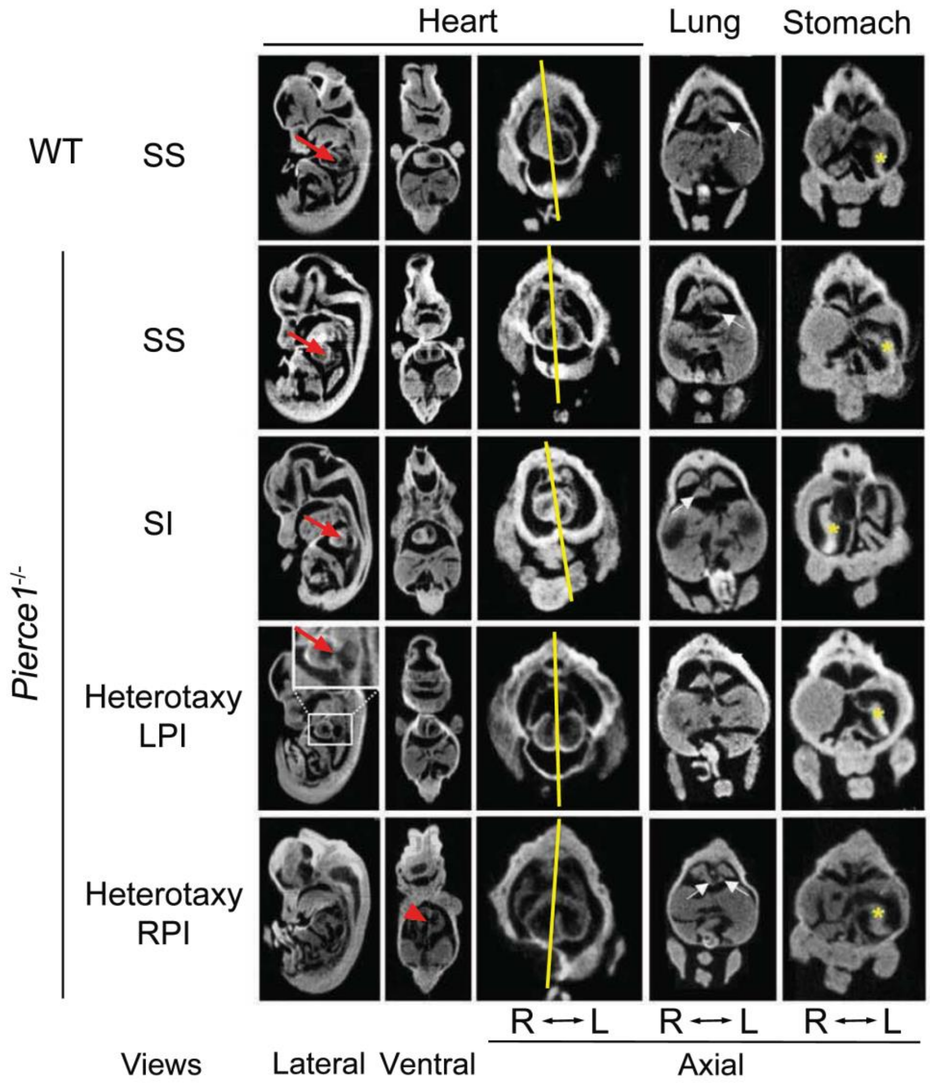

Supplementary Figure S3. Defects in the visceral organs of E13.5 *Pierce1*$^{-/-}$ embryos. Representative micro-computed tomography (μCT) scans of E13.5 wild-type (WT) and *Pierce1*$^{-/-}$ embryos exhibiting *situs solitus* (SS), *situs inversus totalis* (SI), left pulmonary isomerism (LPI), and right pulmonary isomerism (RPI). Normal mitral valve development is identified in *Pierce1*$^{-/-}$ embryos with SS and SI (red arrows). Mitral valve defects (red arrow in the white box) and interventricular septal defects (red arrowhead) are observed in mutant embryos with LPI or RPI. Vertical yellow lines in axial views of the hearts indicate the midline of the dorsoventral axis. Note the position of the postcaval lobe and stomach indicated by the white arrow and asterisk, respectively. R ↔ L indicates the orientation of the mouse organs.

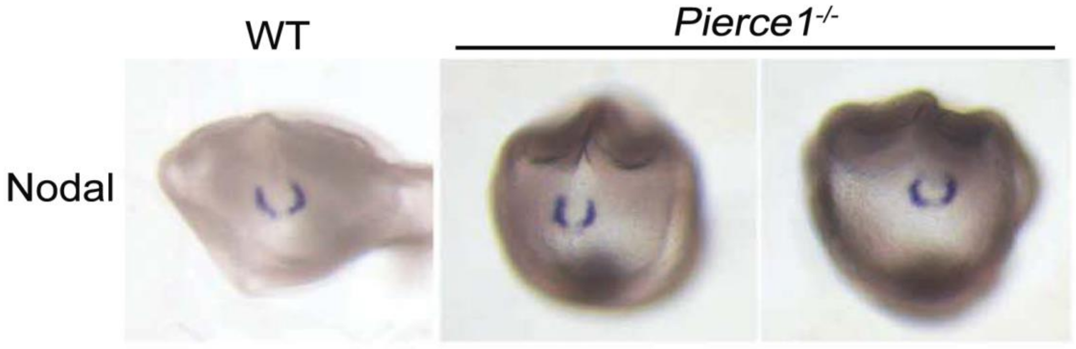

Supplementary Figure S4. Expression of *Nodal* in the perinodal cells of E8.0 wild-type (WT) and *Pierce1*$^{-/-}$ embryos. Expression was detected by whole-mount *in situ* hybridization using a *Nodal* antisense riboprobe. There was no difference in Nodal expression in the perinodal cells of *Pierce1*$^{-/-}$ embryos compared to that of WT embryos.

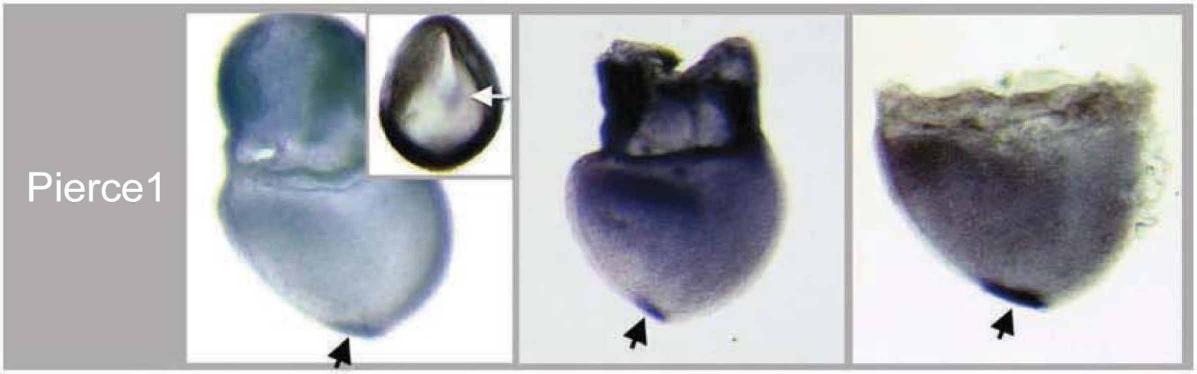

Supplementary Figure S5. Expression of *Pierce1* in the node area of E7.75 - E8.25 wild-type (WT) embryos. Expression was detected by whole-mount *in situ* hybridization using a *Pierce1* antisense riboprobe. Relatively strong expression of *Pierce1* is observed in the node region of WT embryos. All images are lateral views, except for a bottom view shown as an inset in the first panel. Arrows indicate the node region.

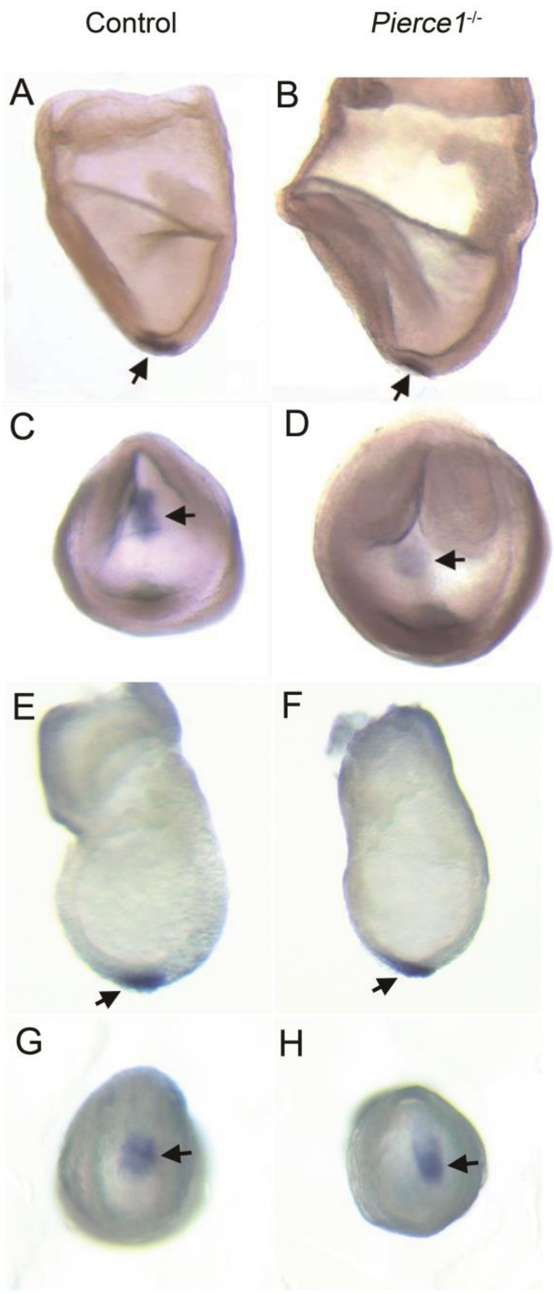

Supplementary Figure S6. Expression of *Dnahc11* and *Noto* in E8.0 wild-type (WT) and *Pierce1*$^{-/-}$ embryos. (**A-H) Expression of** *Dnahc11* (A-D) and *Noto* (E-H) is detected in the node of WT (A, C, E, G) and *Pierce1*$^{-/-}$ **(B, D, F, H)** embryos. Images are a lateral view **(A, B, E, F)** or a bottom view **(C, D, G, H)**. Arrows indicate the node region.

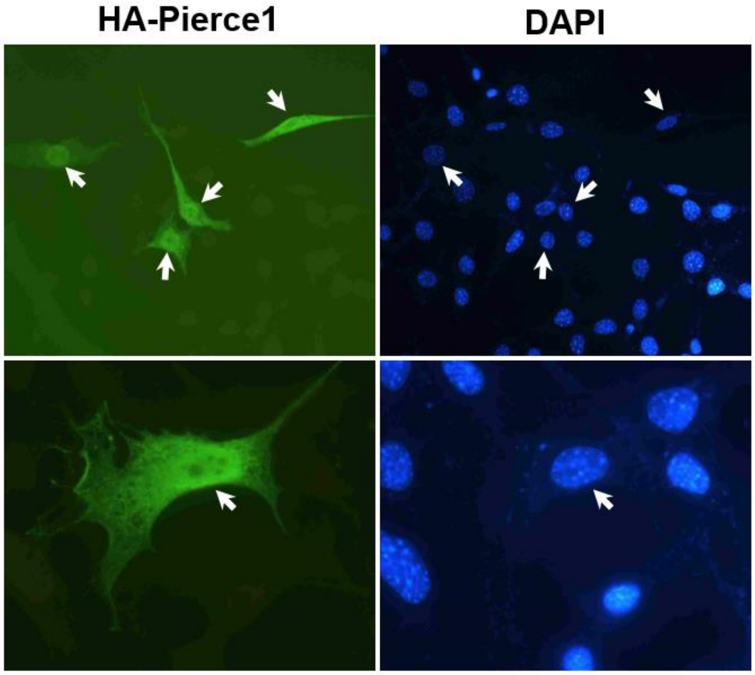

Supplementary Figure S7. Subcellular localization of hemagglutin hemagglutinin (HA)-tagged Pierce1 (HA-Pierce1) that was exogenously expressed in NIH3T3 cells. A construct expressing HA-Pierce1 was transfected into NIH3T3 cells and the overexpressed HA-Pierce1 was cytochemically labeled using an anti-HA antibody as a primary antibody and a fluorescein isothiocyanate (FITC)-labelled secondary antibody. Nuclei were counterstained with 4′,6-diamidino-2-phenylindole (DAPI). Arrows indicate cells expressing HA-Pierce1.

Supplementary Table S1. Partial embryonic lethality induced by *Pierce1* deficiency.

Embryonic Day (E)	*Pierce1*$^{+/+}$	*Pierce1*$^{+/-}$	*Pierce1*$^{-/-}$ Live	*Pierce1*$^{-/-}$ Dead	Total
E13.5	23 (1.00)[a]	41 (1.78)	24 (1.09)	1	89
E14.5	24 (1.00)	50 (2.08)	20 (1.25)	10	104
Adults	52 (1.00)	95 (1.83)	22 (0.42)	0	169

[a]The numbers in parentheses are the ratios of genotypes observed.

Supplementary Table S2. Defects in the specification of LR asymmetry in E13.5 *Pierce1*$^{-/-}$ embryos.

Embryos	Situs[a]	Heart apex	Inferior vena cava	Lung lobation	Stomach position
1	SS	Levocardia	Right	Normal	Left
2	SS	Levocardia	Right	Normal	Left
3	SS	Levocardia	Right	Normal	Left
4	SS	Levocardia	Right	Normal	Left
5	SS	Levocardia	Right	Normal	Left
6	SS	Levocardia	Right	Normal	Left
7	SS	Levocardia	Right	Normal	Left
8	SS	Levocardia	Right	Normal	Left
9	SS	Levocardia[b]	Right	Normal	Left
10	SI	Dextrocardia	Left	Mirror image	Right
11	SI	Dextrocardia[c]	Left	Mirror image	Right
12	Heterotaxia	Mesocardia[d,e]	Left	Left isomerism	Right
13	Heterotaxia	Levocardia[b]	Right	Right isomerism	Left
14	Heterotaxia	Mesocardia[d]	Right	Left isomerism	Left
15	Heterotaxia	Levocardia[b]	Right	Right isomerism	Left
16	Heterotaxia	Levocardia[b]	Right	Right isomerism	Left
17	Heterotaxia	Mesocardia	Left	Left isomerism	Right
18	Heterotaxia	Mesocardia[c,e]	Right	n.d.[g]	Left
19	Heterotaxia	Dextrocardia	Left	n.d.	Right
20	Heterotaxia	Dextrocardia	Left	Normal[h]	Left

[a]SS, *situs solitus*; SI, *situs inversus totalis.*
[b-f]The hearts of E13.5 *Pierce1*$^{-/-}$ embryos show several abnormalities, including interventricular septal defect[b], hypoplastic heart defects[c], mitral valve defects[d], dilated atria[e], and atrophy of the left ventricle[f].
[g]Not determined.
[h]There is no postcaval lobe.

Supplementary Table S3. Mutant mouse models that manifest laterality defects.

Gene	Stage	Situs[a] (%)[b]			Heart[c] (%)			Lung[d] (%)				Abd. organs[e] (%)		Ref
		SS	SI	Het	Lev	Mes	Dex	SS	SI	LPI	RPI	Normal	Affected	
Acvr2b	E18.5 neonate	0	0	100	40	30	30	0	0	0	100	100	0	1
Arl13b	E12.5		n.d[f]		58	0	42	39	6	44	11	52	48	2
Cerl2	neonate >3 weeks		n.d			n.d		64	18	18	9[g]	61	39[g]	3
Cryptic	E18.5 neonate	0	0	100	52	12	36	0	0	0	100	55	45	4
Dnahc5[h]	E16.5-E18.5	24	36	40	37	4	58	29	56	13	3	37	63	5
Dnahc11	neonate	48	39	13	58	0	42	52	39	10	0	52	48	6
Dnaic1	E14.5-E18.5	18	35	47	32	12	56	21	48	31	0	38	62	7
Fgf8	E16.5-P1		n.d			n.d		47	4	0	49	n.d		8
Foxj1	E18.5	20	0	80		n.d		30	10	0	60	40	60	9
Gdf1	neonate	0	0	100		n.d		0	0	0	100	50	50	10
Inv	E18.5		n.d			n.d		25	50	19	6	38	63	9
	E18.5 neonate		n.d			n.d		0	90	10	0	0	100	11
iv	E16.5-E18.5		n.d		45	2	53		n.d			n.d		12
	neonate	36	41	23		n.d		36	41	10	13	n.d		13
Lefty1	neonate	38	0	63		n.d		43	0	58	0	100	0	14
Lefty2[i]	neonate		n.d		94	0	6	100	0	0	0	n.d		15
Mns1	E12.5-E16.5		n.d			n.d		36	36	27	0	n.d		16
Nodal[j,k]	E14.5		n.d		48	0	52	4			88	82	18	17
Noto[l]	E16.5	19	28	53		n.d		19	28	34	13	n.d		18
Nphp3	E9.5-E13.5	28	52	21		n.d			n.d			n.d		19
Pitx2	n.s[m]		n.d			n.d		0	0	0	100	n.d		20 21
Pkd1l1	E13.5-E14.5	0	0	100	75	3	22	0	0	0	100	75	25	22
Pkd2	E13.5-E14.5		n.d		52	7	41	3	0	0	97	63	37	22
	E16.5		n.d		35	27	38	6	4	0	90	35	65	23
Ift88	E9.5	48	52	0		n.d			n.d			n.d		24
Rfx3[h]	E18.5	65	6	29	74	10	16	65	6	26	3[n]	82	18	25
Zic3	E17.5-E18.5	20	4	76		n.d		44	12	8	36	76	24	26

[a]SS, *situs solitus*; SI, *situs inversus totalis*; Het, heterotaxy.
[b]Rounded off to the nearest whole number.
[c]Lev, Levocardia; Mes, mesocardia; Dex, dextrocardia.
[d]LPI, left pulmonary isomerism; RPI, right pulmonary isomerism.
[e]Abd organs, abdominal organs (stomach, spleen, or pancreas).
[f]Not determined.
[g]Among 94 null mice lung phenotype was analyzed in 33 pups died within the first 48 h after birth and abdominal phenotype was analyzed in remaining 61 mice.
[h]Combined data from different experiments.

[i]Mutant embryos with Lefty2-asymmetric enhancer targeted alleles.
[j]Mutant embryos with Nodal hypomorphic alleles.
[k]Ambiguous criterion of the lung situs "lung abnormal asymmetric (8%)" is not included in the lung section.
[l]Ambiguous criterion of the lung situs "lung heterotaxy (6%)" is not included in the lung section.
[m]Not specified.
[n]Two left lung lobes and three right lung lobes.

Supplementary Methods

Micro-computed tomography (μCT). Micro-CT analysis was conducted at Samsung Biomedical Research Institute (Seoul, Korea) as described previously[46]. Briefly, E13.5 embryos were harvested, fixed in 10% buffered formalin (Sigma), and stained to saturation in a solution of 0.1 M sodium cacodylate (pH 7.2, Sigma), 1% glutaraldehyde (Sigma), and 1% osmium tetroxide (Sigma) by incubating at room temperature. Embryos were successively washed in 0.1 M sodium cacodylate buffer and phosphate-buffered saline (PBS) and dehydrated through a series of ethanol gradients prior to scanning. Volumetric CT scans of embryos were performed at 20-μm isometric voxel resolution using an Inveon positron emission tomography (PET)/CT system (Siemens Healthcare Global). Images were reconstructed with Amide's Medical Image Data Examiner (AMIDE) software[27].

Cytochemical analysis.

NIH3T3 cells were cultivated on gelatin-coated glass coverslips and the expression construct of HA-Pierce1 was transfected into the cells as previously described[28,29]. Briefly, Transfection was performed with the LipofectAMINE PLUS reagent according to the manufacturer's instructions (Invitrogen). The cells were fixed by 3.7% paraformaldehyde (Sigma-Aldrich) for 15 min at room temperature and washed in phosphate-buffered saline (PBS). The fixed cells were permeabilized with 0.5% Triton X-100 (Amresco) in PBS for 5 min and then incubated in a blocking solution [10% normal goat serum (Vector Laboratories) and 0.1 % gelatin in PBS] for 30 minutes at room temperature. The cells were subsequently stained with a primary antibody

(anti-HA rabbit antibody, Santa Cruz Biotech) and then with a fluorescein isothiocyanate (FITC)-labelled secondary antibody (Jackson ImmunoResearch) diluted in the blocking solution for 1 hour and 30 minutes, respectively, in a humidified chamber at room temperature. After counterstaining with 4′,6-diamidino-2-phenylindole (DAPI), coverslips were mounted on the slideglass using Gel Mount (BioMeda Corporation). Fluorescence microscopy was conducted using Axioskop 2 equipped with Axiophot II (Carl Zeiss Vision) and the results were analyzed with AxioVision software (Carl Zeiss Vision).

Supplementary References

1. Oh, S. P. & Li, E. The signaling pathway mediated by the type IIB activin receptor controls axial patterning and lateral asymmetry in the mouse. *Genes Dev* **11**, 1812–1826 (1997).
2. Larkins, C. E., Long, A. B. & Caspary, T. Defective Nodal and Cerl2 expression in the Arl13b(hnn) mutant node underlie its heterotaxia. *Dev Biol* **367**, 15–24 (2012).
3. Marques, S., Borges, A. C., Silva, A. C., Freitas, S., Cordenonsi, M., & Belo, J. A. The activity of the Nodal antagonist Cerl-2 in the mouse node is required for correct L/R body axis. *Genes Dev* **18**, 2342–2347 (2004).
4. Yan, Y. T. *et al.* Conserved requirement for EGF-CFC genes in vertebrate left-right axis formation. *Genes Dev* **13**, 2527–2537 (1999).
5. Tan, S. Y. *et al.* Heterotaxy and complex structural heart defects in a mutant mouse model of primary ciliary dyskinesia. *J Clin Invest* **117**, 3742–3752 (2007).
6. Supp, D. M. *et al.* Targeted deletion of the ATP binding domain of left-right dynein confirms its role in specifying development of left-right asymmetries. *Development* **126**, 5495–5504 (1999).
7. Francis, R. J. B., Christopher, A., Devine, W. A., Ostrowski, L. & Lo, C. Congenital heart disease and the specification of left-right asymmetry. *Am J Physiol Heart Circ Physiol* **302**, H2102–H2111 (2012).
8. Meyers, E. N. & Martin, G. R. Differences in left-right axis pathways in mouse and chick: functions of FGF8 and SHH. *Science* **285**, 403–406 (1999).
9. Tamakoshi, T. *et al.* Roles of the Foxj1 and Inv genes in the left-right determination of internal organs in mice. *Biochem Biophys Res Commun* **339**, 932–938 (2006).
10. Rankin, C. T., Bunton, T., Lawler, A. M. & Lee, S. J. Regulation of left-right patterning in mice by growth/differentiation factor-1. *Nat Genet* **24**, 262–265 (2000).
11. Morishima, M. *et al.* Situs variation and cardiovascular anomalies in the transgenic mouse insertional mutation, inv. *Teratology* **57**, 302–309 (1998).
12. Icardo, J. M. & Vega, M. J. S. de. Spectrum of heart malformations in mice with situs solitus, situs inversus, and associated visceral heterotaxy. *Circulation* **84**, 2547–2558 (1991).
13. Oh, S. P. & Li, E. Gene-dosage-sensitive genetic interactions between inversus viscerum (iv), nodal, and activin type IIB receptor (ActRIIB) genes in asymmetrical patterning of the visceral organs along the left-right axis. *Dev Dyn* **224**, 279–290 (2002).
14. Meno, C. *et al.* lefty-1 is required for left-right determination as a regulator of lefty-2 and nodal. *Cell* **94**, 287–297 (1998).
15. Meno, C. *et al.* Diffusion of nodal signaling activity in the absence of the feedback inhibitor Lefty2. *Dev Cell* **1**, 127–138 (2001).
16. Zhou, J., Yang, F., Leu, N. A. & Wang, P. J. MNS1 is essential for spermiogenesis and motile ciliary functions in mice. *PLoS Genet* **8**, e1002516 (2012).
17. Lowe, L. A., Yamada, S. & Kuehn, M. R. Genetic dissection of nodal function in patterning the mouse embryo. *Dev Camb Engl* **128**, 1831–1843 (2001).
18. Beckers, A., Alten, L., Viebahn, C., Andre, P. & Gossler, A. The mouse homeobox gene Noto regulates node morphogenesis, notochordal ciliogenesis, and left–right patterning.

Proc Natl Acad Sci **104**, 15765–15770 (2007).
19. Bergmann, C. *et al.* Loss of nephrocystin-3 function can cause embryonic lethality, Meckel-Gruber-like syndrome, situs inversus, and renal-hepatic-pancreatic dysplasia. *Am J Hum Genet* **82**, 959–970 (2008).
20. Kitamura, K. *et al.* Mouse Pitx2 deficiency leads to anomalies of the ventral body wall, heart, extra- and periocular mesoderm and right pulmonary isomerism. *Dev Camb Engl* **126**, 5749–5758 (1999).
21. Lin, C. R. *et al.* Pitx2 regulates lung asymmetry, cardiac positioning and pituitary and tooth morphogenesis. *Nature* **401**, 279–282 (1999).
22. Field, S. *et al.* Pkd1l1 establishes left-right asymmetry and physically interacts with Pkd2. *Dev Camb Engl* **138**, 1131–1142 (2011).
23. Pennekamp, P. *et al.* The ion channel polycystin-2 is required for left-right axis determination in mice. *Curr Biol CB* **12**, 938–943 (2002).
24. Murcia, N. S. *et al.* The Oak Ridge Polycystic Kidney (orpk) disease gene is required for left-right axis determination. *Development* **127**, 2347–2355 (2000).
25. Bonnafe, E. *et al.* The transcription factor RFX3 directs nodal cilium development and left-right asymmetry specification. *Mol Cell Biol* **24**, 4417–4427 (2004).
26. Purandare, S. M. *et al.* A complex syndrome of left-right axis, central nervous system and axial skeleton defects in Zic3 mutant mice. *Dev Camb Engl* **129**, 2293–2302 (2002).
27. Loening, A. M. & Gambhir, S. S. AMIDE: a free software tool for multimodality medical image analysis. *Mol. Imaging* **2,** 131–137 (2003).
28. Sung, Y. H., Kim, H. J. & Lee, H.-W. Identification of a novel Rb-regulated gene associated with the cell cycle. *Mol. Cells* **24,** 409–415 (2007).
29. Sung, Y. H. *et al.* Pierce1, a Novel p53 Target Gene Contributing to the Ultraviolet-Induced DNA Damage Response. *Cancer Res.* **70,** 10454–10463 (2010).
